# Self-Help in Institutional Economy

**Published:** 1913-01-04

**Authors:** 


### SELF-HELP IN INSTITUTIONAL ECONOMY.

### III.—Do Hospital Dairies Pay?

Of all the provisions contracted for from outside sources the one which demands the closest scrutiny on the part of the hospital authorities is undoubtedly milk. It is a fluid most easily adulterated and most readily contaminated by impurities. When adulterated it loses from 10 to 50 per cent. of its food value. When contaminated with organic impurities it becomes an active source of danger and a potential cause of disease. Contamination and adulteration are both easily guarded against with proper care, and yet an investigation of the milk supply of a number of hospitals recently made has shown that both are by no means infrequently met with in the milk supplied to such institutions. Out of twenty cases, the milk was found absolutely pure in only ten; in three it was adulterated by the admixture of water; in five it contained an undue amount of preservative; in seven it was not bacteriologically pure; in four it contained a diminished quantity of fat. The specimens from which these figures of the analyses were taken were portions of the ordinary milk supplied to well-known institutions under tender or contract. It will thus be seen that even in cases where certain precautions are taken it is not always possible to ensure the purity of the provisions supplied if they happen to be of a kind so easily contaminated or adulterated as milk. It may be argued that milk adulteration is one of the easiest frauds to detect, since it can be discovered by the use of a simple lactometer in most cases. That is true enough, but how many institutions possess and employ as a routine test this simple instrument? Ordinarily the milk is obtained from a "reputable dairy," and the superintendent trusts that the daily supply will never vary in quality as it will never vary in quantity. This trust very often is false, as the figures of the analyses above quoted show.

Obviously the only certain way in which hospitals can assure themselves that the milk supplied to them is absolutely pure, without going to the expense of elaborate bacteriological examination, is to obtain the liquid from a dairy which is entirely above suspicion. Some institutions have, therefore, attempted to deal with the problem of a safe milk supply by establishing their own dairies. This policy ensures the purity of the milk and enables the hospital to know what sort of cows yield it, and under what conditions it is obtained—points, be it noted, that cannot be estimated by analysis. When the contractor supplies the milk there is no way, short of inspection of his dairies by a competent veterinary surgeon, of telling whether the cows are healthy or not. Tuberculosis is a very common disease with dairy cattle, and, although the danger of drinking milk derived from such cows has probably been greatly over-estimated, it is nevertheless desirable that such milk should be scrupulously eliminated from the hospital milk room. Another important point is the condition of the dairy and the manner in which the milk is obtained. To be absolutely safe as regards all these points the hospital authorities must be satisfied that the dairy whence they obtain their supply of milk is up to date and managed in accordance with the rules of hygiene.

In Continental hospitals several interesting "hospital dairies" have been established of late years. Model dairies exist at Düsseldorf Hospital and in connection with the University Hospital at Marburg.* The stables are simple, plain, whitewashed structures kept scrupulously clean, a new coat of whitewash being given to walls and ceiling at least once every month, while special arrangements are made to keep the stalls free from dung. The cows are tested periodically, the tuberculin test being employed. Every possible precaution is taken to prevent contamination of the milk. Before milking, the teats and udders of the cows are cleansed with warm water and dried with soft cloths: the milker (unless an apparatus is employed) sterilises his hands by careful washing with soap and water, and all dairy utensils are sterilised by washing in boiling water, the buckets being actually boiled in a large steriliser. Half an hour before the milking everything is carefully arranged: cleaning of the stalls and of the animals is only allowed after the milking. The buckets are covered with sterilised gauze, and when filled are carried at once into the cooling chambers, where the milk is carefully transferred to sterile and hermetically sealed "transport flasks," in which it is conveyed to the hospital.† There is a well-equipped bacteriological laboratory in connection with the dairy. Here a specimen of milk from every animal is daily carefully examined, cultures being taken on Petri plates. Every month an analysis of the cream and centrifugalised deposit is made by the Gerber method to discover if tubercle bacilli are present, while regular chemical analysis to estimate the amount of fat present are performed. Owing to the fact that the best conditions for dairy work and the necessary field and meadow for the cows are only obtainable outside the city itself, the dairy is located at a considerable distance from the hospital. This makes it necessary to employ special

* An interesting and detailed account of the dairy at this latter institute appeared in the *Zeitschrift für soziale Medizin*, 1909. The institution does not possess its own cows.

† These elaborate aseptic precautions must not be looked upon as superfluous. Before the Düsseldorf Municipal Hospital possessed its own dairy it contracted for its milk supply, and the milk was found to contain, on an average, 25,000 to 30,000 germs per cubic centimetre. As a comparison, it may be stated that a recently analysed sample of milk supplied to a London hospital contained an average of 38,000 germs per c.c. The milk supplied by the Düsseldorf Municipal Hospital Dairy contains on an average 5,000 to 7,000 germs per c.c., although a maximum "germ content" of 18,000 is looked upon as normal. A large amount of valuable bacteriological research work has been done in connection with the dairy, and the results are of such practical importance that their consideration is recommended to all hospital workers.

quick transport methods. Where the railway is available this has been used, but preference is now given to motor transport. The milk motors are specially constructed with a cool chamber and an ice chamber, in which the milk can be safely kept for some time. It is found that by this means the milk is kept pure and in the best condition for subsequent use.

Does a private dairy for a hospital pay? The Düsseldorf figures do not show that the new department is as successul as the hospital butcher's department—there is as yet no excess of income over expenditure. During the first two years the accounts showed that the expenditure was far in excess of the income, and it was doubted whether the new department justified its existence. Owing to the high initial cost the first year's expenditure was far beyond that of any subsequent year. It must be remembered that an important item of the expenditure is the money spent in purchasing the cattle; the interest on this money, which may be reckoned as so much capital, must be added to the annual expenditure. In addition there is the comparatively expensive upkeep of the bacteriological laboratory and its staff and of the motor transport service, besides lease of grazing land and upkeep of stables and cowhouses. Yet, notwithstanding all this, the experiment has proved a success, for at present income balances expenditure, and the hospital gets the benefit of absolutely pure milk obtained under model conditions. Zeidler, writing in 1911, remarks that the department "is of incalculable value, particularly since it enables many as yet unsolved problems in connection with the milk supply to be investigated, and since it gives a model example to every private dairy-owner how a dairy should be managed under the most sanitary and hygienic conditions." We have already referred to the important asset which a hospital possesses in such a dairy, in that it is able to guarantee the purity of the milk and the cleanliness of the methods by which that milk is obtained.

Co-operation, so far as the milk supply is concerned, has not yet been attempted. It seems, however, that in this matter of milk supply such co-operation between two or more large hospitals would be productive of the best results, and well worth a trial.

